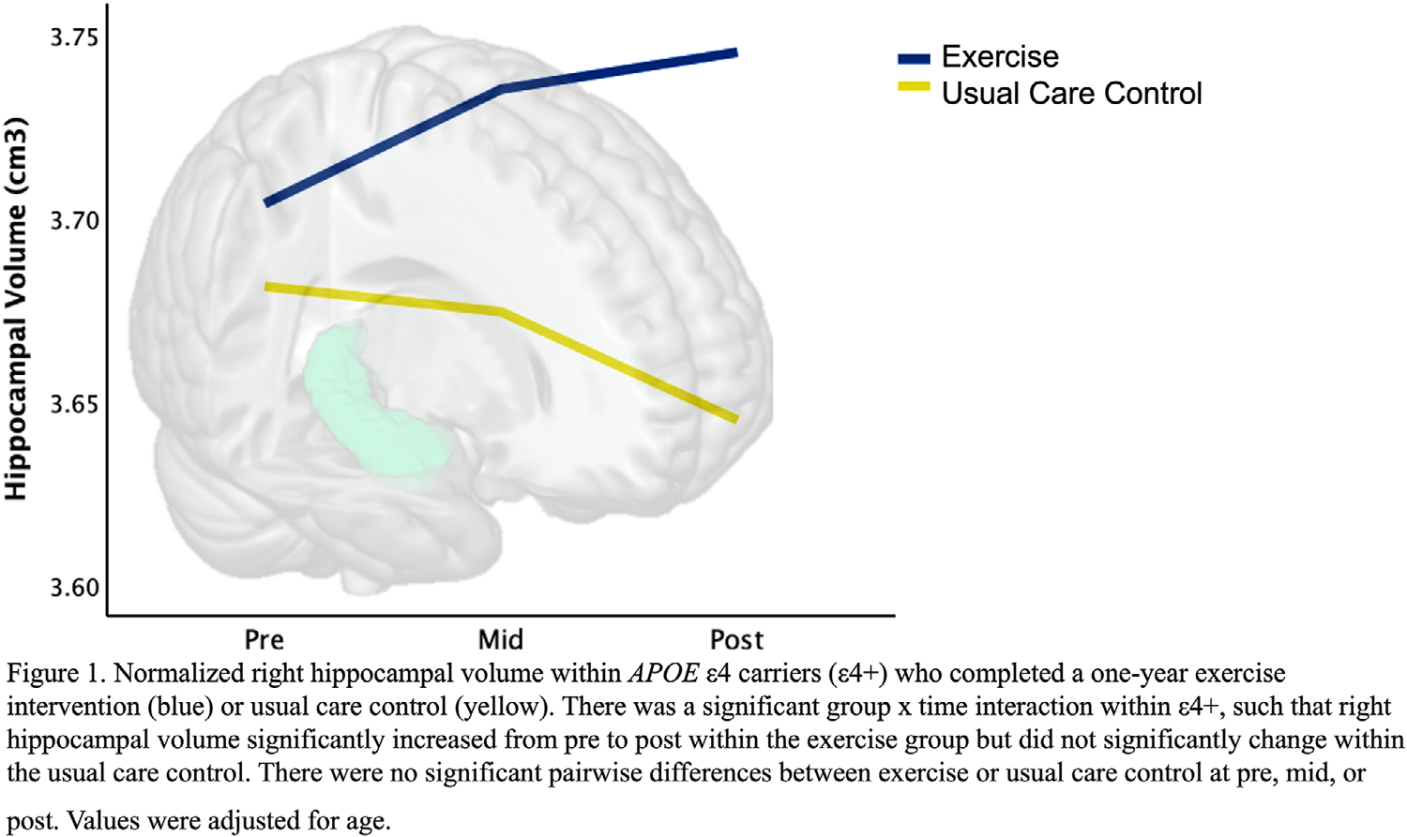# The effect of exercise and ApolipoproteinE ε4 on hippocampal volume in middle‐aged sedentary adults with heightened risk for Alzheimer’s disease

**DOI:** 10.1002/alz.095534

**Published:** 2025-01-09

**Authors:** Alexis B Slutsky‐Ganesh, Jennifer L Etnier, Samantha L DuBois, Jarod C Vance, Kyoung Shin Park, Emily Bechke, Hadassah Som‐Pimpong, Megan M O'Brokta, Delaney Thibodeau, Aiko Ueno, William B Karper, Brittany D Armstrong, Jeffrey D Labban, Christina E Hugenschmidt, Laurie Wideman

**Affiliations:** ^1^ Emory University, Atlanta, GA USA; ^2^ University of North Carolina at Greensboro, Greensboro, NC USA; ^3^ Wake Forest School of Medicine, Winston‐Salem, NC USA

## Abstract

**Background:**

ApolipoproteinE ε4 carriers (ε4+) have a heightened risk for Alzheimer’s disease (AD) and experience accelerated hippocampal volume (HV) loss during older adulthood, which tends to precede cognitive decline. Exercise interventions can increase HV in sedentary older adults, however it’s currently unknown how exercise affects HV in sedentary middle‐aged adults and if ε4 carrier status moderates this relationship.

**Methods:**

A subset of 44 adults from an ongoing randomized controlled clinical trial (NIH/NIA: R01AG058919) completed baseline (pre), 6‐month (mid), and 12‐month (post) testing during a one‐year exercise intervention (n = 29, 52% ε4+, 5 males, Age_mean_ = 55.05±6.20) or usual care control (control; n = 15, 53% ε4+; 3 males, Age_mean_ = 60.27±3.65). Participants were cognitively normal, sedentary, and had a family history of AD (FH+). Genetic sampling was completed at pre‐test via passive drool. Structural neuroimaging was completed at pre‐, mid‐, and post‐test. Brain segmentation was completed with Freesurfer’s automated hippocampal longitudinal pipeline (v7). Left and right HVs were extracted and normalized based on intracranial volume. Group (exercise, control) x time (pre, mid, post) x carrier status (ε4+, ε4‐) interactions were examined with mixed model analysis of covariance for the left and right HV, controlling for age. Posthoc examinations included group x time interactions within ε4+ and ε4‐ and follow‐up pairwise comparisons.

**Results:**

There were significant three‐way interactions for left (F_(1,39)_ = 8.76, *p* = .005) and right (F_(1,39)_ = 13.48, *p*<.001) HV. Follow‐up group x time interactions within ε4‐ were not significant, but were significant within ε4+ for the right HV (F_(1,19)_ = 6.40, *p* = .02) but not the left HV (F_(1,19)_ = 4.27, *p* = .05). ε4+ exercisers increased right HV from pre‐to‐post intervention (mean change = 0.41cm^3^, *p* = .02); no other tests were significant.

**Conclusion:**

Exercise‐related benefits for HV for sedentary cognitively‐normal, middle‐aged adults with a FH+ are such that right HV increased following one‐year of exercise in ε4+ but not in ε4‐. Though exercise generally increases HV in older adults, during middle age the effects may be limited to a vulnerable population such as ε4+ who tend to have smaller HV relative to ε4‐ throughout adulthood. The content of this abstract is solely the responsibility of the authors and does not necessarily represent the official views of the NIH.